# DETERMINANTS OF LEISURE-TIME PHYSICAL ACTIVITY IN OLDER, RURAL CANCER SURVIVORS IN CENTRAL PENNSYLVANIA

**DOI:** 10.1093/geroni/igz038.1424

**Published:** 2019-11-08

**Authors:** Scherezade K Mama, Nishat Bhuiyan, Eugene Lengerich, Kathryn Schmitz

**Affiliations:** 1 The Pennsylvania State University, University Park, Pennsylvania, United States; 2 Pennsylvania State University, Hershey, Pennsylvania, United States; 3 The Pennsylvania State University College of Medicine, Hershey, Pennsylvania, United States

## Abstract

This study explored social and environmental determinants of leisure-time physical activity (LTPA) in cancer survivors (CS) residing in Central Pennsylvania, a largely rural region. Rural CS completed questionnaires assessing LTPA, social support (SS) for LTPA, home and neighborhood environments for LTPA. Logistic regression models were used to assess associations with being active/inactive. Participants (n=219) were categorized as mature survivors (<75 years, 80.7%) or elderly survivors (>=75 years, 19.3%). Only 28.2% of elderly survivors reported meeting LTPA guidelines compared to 45.6% of mature survivors. Survivors reporting SS for LTPA were 10% more likely to active than those who did not have SS (OR=1.1, CI 1-1.1). Mature survivors that reported environmental support (home: OR=1.2: CI 1-1.3; neighborhood: OR=1.8, CI: 1-3.2) were more likely to be active than those without strong environmental support. Creating more supportive environments to foster LTPA in elderly survivors in rural areas is a key priority for future research.

